# Supplementary material for: Predictive factors involving the remission and recurrence of hypertension post-laparoscopic sleeve gastrectomy in Japanese patients with severe obesity
Source: PLoS One. 2024 Dec 17;19(12):e0314792. doi: 10.1371/journal.pone.0314792 (PMC11651579; doi:10.1371/journal.pone.0314792)
Supplement: S1 Table — (DOCX) [file pone.0314792.s001.docx]

S1 Table Relationship between plasma aldosterone concentration and type 2 diabetes-related parameters

|  | Variable | Baseline | | 6 months after LSG | | 12 months after LSG | | 36 months after LSG | |  |
| --- | --- | --- | --- | --- | --- | --- | --- | --- | --- | --- |
|  |  | ρ | P value | ρ | P value | ρ | P value | ρ | P value |  |
|  | HbA1c (%) | 0.023 | 0.854 | -0.236 | 0.065 | -0.227 | 0.093 | -0.410 | **0.024** |  |
|  | HOMA-IR (no unit) | 0.117 | 0.368 | 0.195 | 0.133 | 0.182 | 0.192 | -0.188 | 0.329 |  |
|  | HOMA-β (no unit) | 0.003 | 0.979 | 0.322 | **0.012** | 0.230 | 0.093 | 0.128 | 0.518 |  |

HbA1c, hemoglobin A1c; HOMA-IR, homeostasis model assessment of insulin resistance; HOMA-β; homeostasis model assessment of beta cell function.

P values < 0.05 are shown in bold. Correlation analysis was performed using Spearman's rank correlation coefficient.
