# Supplementary material for: Predictive factors involving the remission and recurrence of hypertension post-laparoscopic sleeve gastrectomy in Japanese patients with severe obesity
Source: PLoS One. 2024 Dec 17;19(12):e0314792. doi: 10.1371/journal.pone.0314792 (PMC11651579; doi:10.1371/journal.pone.0314792)
Supplement: Attachment — Submitted filename: Response_to_Reviewers_2.docx [file pone.0314792.s003.docx]

Prof. Emily Chenette

PLOS ONE, Editor in Chief

Dear Prof. Chenette,

The comments of the reviewers have been very helpful for us to revise our manuscript. We have attempted to address the questions raised by the reviewers. Our point-by-point responses to the reviewers’ comments are provided below.

Reviewer #3: Dear authors,

Thanks for your responses. The manuscript is much better now.

Please revise:

- In the abstract, line 22: "LSG in patients who are severely obese," change for LSG in patients who have severe obesity.

Reply:

Thank you again for your kind and constructive suggestions. As you suggested, the wording has been corrected.

- Instead of weight regain, use the IFSO definition: Recurrent weight gain.

Reply：

As suggested, we have changed the word "weight regain" to "recurrent weight gain" in the manuscript in accordance with the IFSO definition.

Sincerely,

Hideki Kumagai

Department of Surgery, School of Medicine, Iwate Medical University

2-1-1 Idaidori, Yahaba-cho, Shiwa-gun, Iwate, 028-3695, Japan.
